# Specialised Teachers’ Perceptions on the Management of Aggressive Behaviours in Children and Adolescents with Autism Spectrum Disorders

**DOI:** 10.3390/ijerph17238775

**Published:** 2020-11-26

**Authors:** Miran Jung, Eunmi Lee

**Affiliations:** 1 Department of Nursing, Baekseok University, Cheonan 31065, Korea; rcuty@bu.ac.kr; 2 Department of Nursing, Research Institute for Basic Science, Hoseo University, Asan 31499, Korea

**Keywords:** autism spectrum disorder, ASD, autism, childhood, aggressive behavior

## Abstract

This study aimed to explore and describe the perception of specialized teachers regarding the management of aggressive behaviors in children and adolescents with autism spectrum disorders. Data were collected from 13 specialized teachers working in primary and secondary schools, using focus group interviews, and interview data were analyzed using an open coding method. The analysis of the specialized teachers’ perceptions of the management of aggression in children and adolescents with autism revealed the following results. A central theme “consistent practices to smooth edges” was conceptualized along with the categories: educational responses to individual behaviors, which had sub-themes of “identification of aggressive behavior patterns” and “strategic responses to aggressive behaviors observed”; experience in interventions for aggressive behaviors, with sub-themes of “individual intervention practices” and “school-led therapeutic support” and “factors preventing mitigation of aggression”; and acceptance of virtual reality (VR) based intervention model for aggression, with sub-themes of “acceptance of VR-based program applications” and “proposal for VR-based program contents.” Based on the specialized teachers’ perceptions examined in this study, more effective education and training intervention programs and support systems can be developed and provided for the management of aggressive behaviors in children and adolescents with autism.

## 1. Introduction

Autism spectrum disorder (ASD) is a common neurodevelopmental disorder characterized by persistent deficits in communication, social interaction, activities, and restricted interests [1]. In addition to impairments in communication and social interaction and the presence of restricted, repetitive, or stereotyped behavior patterns, ASD can have different symptoms related to behavior, social skills, preferences, physical performance, and learning ability; failing to meet developmental milestones is required for a diagnosis of autism [2].

Humans are social animals. They establish social relations with others through human–environment interactions; thus, deficits in social interactions make it difficult for people with autism to fit socially. Specifically, they may develop insecure attachments or be prone to aggressive behavior as a consequence of being socially disadvantaged [3]. 

Aggression is generally defined as physical and verbal behavior that is intended to cause harm to others, threatening self-defense attitudes, and even thoughts and emotions evoked by such intentions [4]. ASD is not itself a violent disorder, and not all people with autism are more aggressive than those who do not have ASD. Nonetheless, children with ASD are reported to have more aggressive behaviors when compared to children with intellectual disabilities or Down syndrome. Moreover, in the presence of other mental health disorders, ASD can cause dramatic changes in behavior [5,6,7]. Aggressive behavior appears when critical thinking does not occur properly during complex cognitive processes, and is highly influenced by social and cognitive skills [8]. Hence, interventions for aggressive behaviors in children and adolescents with ASD are critical, not only to improve their social interactions, but also to keep them safe and protected. 

Aggressive behaviors in children and adolescents with ASD are described as maladaptive behaviors, involving a combination of anger, hostility, vigilance, and anxiety, and are divided into extraverted and introverted aggression. Extraverted aggression acts as an obstacle to social interactions as it manifests in destructive or harmful behaviors, whereas introverted aggression manifests through behaviors like self-harm, truancy, and lying. Therefore, parents and teachers have difficulties in dealing with the challenging behaviors of children and adolescents with autism, and take actions to control their behaviors in an effort to reduce negative outcomes. However, such an attempt to prevent or control the aggressive behavior of people with autism may have the opposite of the intended effect, thereby calling for more therapeutic interventions. 

Previous studies have proposed a variety of interventions for the management of aggressive behaviors in children and adolescents with autism, and many of them were developed with a focus on the role of specialized teachers and applied to school settings. These studies have explored various educational approaches for teachers specialized in teaching children with autism, and developed integrated models that address the individual needs of these children. The important role of specialized teachers has also been underscored [9,10,11,12,13,14]. However, some specialized teachers were found to lack confidence in working with children with ASD due to a lack of preparation, leading to a decline in teaching effectiveness [15]. In a Greek study, special education teachers were confused about their teaching methods, although they had better knowledge of ASD than general education teachers [16]. Interventions for aggressive behaviors of people with ASD have used a behavioral focus to improve safety skills and education in real-world settings. Meanwhile, advanced VR technology has led to increasing research on VR-based training and applications of simulation content. Compared with VR-based training, repetitive education in real-world settings is reported to be less stimulating and interesting, in order to keep children with ASD immersed in learning [17,18,19]. Hence, exploring a new approach to aggression management in children and adolescents with autism, based on specialized teachers’ perceptions and intervention practices, is worthwhile. In this study, we conducted semi-structured interviews to identify specialized teachers’ perceptions of the management of aggressive behaviors of children and adolescents with ASD, with the ultimate aim of providing implications for the development of aggression management training programs and improvements in teacher competence.

## 2. Materials and Methods 

### 2.1. Study Design

This study used a qualitative descriptive design to gain insights from specialized teachers regarding therapeutic interventions for aggressive behaviors in children and adolescents with ASD. Data were collected through a focus group interview process, which is known to be an effective technique when identifying the perceptions of a specific group about a phenomenon [20].

### 2.2. Study Participants 

This study included a total of 13 specialized teachers working at primary and secondary schools in Seoul and surrounding cities. Participants were screened based on the inclusion criteria: teachers educating children and adolescents with ADS with more than six months of experience, informed about the objectives of the study, and encouraged to express their views freely. Those who were taking leave, and those who met students 1–2 times a week were excluded. 

Initially, 15 participants were recruited via the websites visited by specialized teachers in Seoul and surrounding cities, from 5 primary schools, middle schools, and high schools. Of those, one primary school teacher and one middle school teacher were excluded because they did not turn up for the interview. The remaining 13 teachers were included in the study. The demographic characteristics of the participants are presented in Table 1. 

### 2.3. Data Collection

Data were collected through semi-structured focus group interviews from 26 February to 15 March 2020.

#### 2.3.1. Development of Interview Questions

Two investigators drafted a set of interview questions based on the literature that was reviewed for the validation test and subsequent discussion. The drafted questions were reviewed by two professors with experience conducting autism-related research or focus group interviews in qualitative research for modification. The main themes of the questions included (1) aggressive behaviors of children and adolescents with ASD, (2) strategic responses to aggressive behaviors observed, (3) experience in interventions for aggressive behaviors, (4) mitigation of aggression in these children and adolescents, and factors hindering aggression mitigation; and (5) application of VR-based interventions. The semi-structured interview questions were presented by a moderator in an open-ended format during the interview. 

#### 2.3.2. Interview Process 

Thirteen specialized teachers were divided into three groups according to school type: primary school (*n* = 4), middle school (*n* = 4), and high school (*n* = 5). Before the interview started, participants were fully briefed about the objectives of the study and the process of conducting the focus group discussions, and they provided written informed consent. 

Group discussions were conducted by a professional, external moderator, while the participants were encouraged to express their opinions as freely as possible. If the participants’ responses were vague and insufficient regarding the presented topic, additional questions were asked to elicit more precise responses. One investigator recorded the entirety of each group interview and took notes to capture nonverbal information, such as group atmosphere and nonverbal communication between participants. In addition, the total number of opportunities to speak was pre-set to allow each person to have an equal opportunity to share their insights. 

Separately, the participants were asked to share their recent experiences in the application of VR-based intervention programs for aggression mitigation. In addition to experiences, they were also instructed to share all things necessary to meet the requirements and needs, and to achieve improvements, in relation to VR-based interventions. Most of the participants were involved in applying VR-based interventions, directly or indirectly, and they provided information that needs to be further explored in future studies. 

Interviews were conducted at a conference room in Seoul, and participants sat facing each other around a U-shaped table. Each group interview took about 90–100 min. 

#### 2.3.3. Focus Group Transcription

One of the investigators primarily transcribed the discussion during focus group interviews using her notebook, and the transcript was finalized by checking focus group recordings and field notes repeatedly to include nonverbal information, such as group atmosphere and participants’ facial expressions. The interview transcription, prepared using Microsoft Word Version 2019, was 111 pages long in A4 page size (font size: 10, line spacing: 1.0). 

### 2.4. Statistical Analysis

To determine the perceptions of the specialized teachers on the management of aggressive behaviors in children and adolescents with ASD, interview data were analyzed using the open coding method that was developed by Strauss and Corbin (1998) [21]. Open coding is an analytic process in which specific concepts are derived from the observed data, and the properties and dimensions of the concepts are defined. To achieve open coding, we broke down the data in the interview transcripts into words, sentences, paragraphs, and lines, and deeply analyzed them to grasp the meaning and concept of each segment through repeated reading of the data prepared. Through this process, a total of 83 concepts were first extracted. After that, the extracted data were revised and supplemented through expert advice, and as a result of two more reviews among researchers, a total of 71 concepts were extracted secondarily. In the second step, concepts were categorized by comparing the properties of the segmented parts with respect to similarities and differences. The coding process yielded one core theme, and three categories, as well as sub-themes. 

### 2.5. Measures to Ensure Reliability 

Reliability in qualitative research refers to the extent to which the research findings represent the actual experience of research participants in the social context [22]. To establish the credibility of this study, the two investigators deeply analyzed interview data (video recordings and transcripts) through repeated readings, sought consultation from two professors who have experience in conducting focus group research, and carried out discussions to reach inter-investigator agreement regarding the core theme, categories, and sub-themes. 

### 2.6. Ethical Consideration

Prior to the start of research, this study was reviewed and approved by the Institutional Review Board of University B (Approval number: BUIRB-202002-HR-027). All participants were informed about the objectives of the study, anonymous treatment of data, interview data recording and taking notes, exclusive data use for research only, the option to leave the study at any time, and provided written informed consent if they wanted to take part in the study. During this process, the participants were given sufficient time to read the consent document before signing it. Two copies were made of the consent document; one was given to the participant and one was retained by the investigator.

## 3. Results

**Figure 1:**
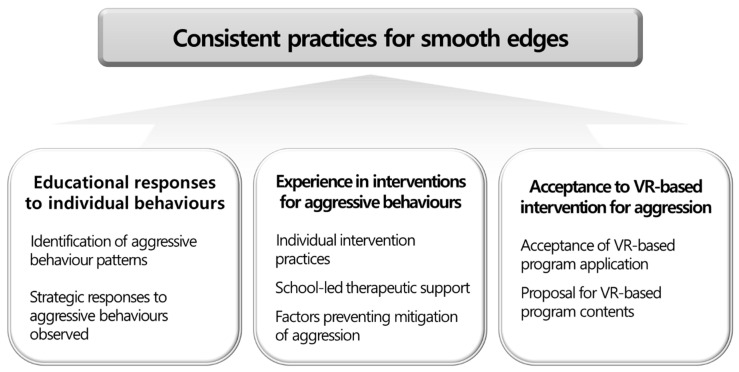
Core theme, categories, and sub-themes through qualitative analysis.

Qualitative analysis of interview data yielded one core theme, three categories, and eight sub-themes as follows. The core theme is ”consistent practices to smooth edges“ while the categories are: (1) educational responses to individual behaviors, (2) experience in interventions for aggressive behaviors, and (3) acceptance of VR-based interventions for aggression in children and adolescents with ASD. The relationships between these themes are presented in Figure 1.

### 3.1. Core Theme: “Consistent Practices to Smooth Edges”

The analysis of the participants’ perceptions of aggressive behaviors in children and adolescents with ASD revealed that most of these students did not exhibit aggressive behavioral patterns; nonetheless, the characteristics of their aggressive behaviors needed to be alleviated or discontinued. The participants agreed that aggressive behaviors cannot be controlled through one-time management and that a consistent and constant treatment approach was required for the behaviors to slowly decline and discontinue. They also said that such a treatment process would be difficult to continue for all the people involved, but that it could not be avoided. In total, the core theme of the participants’ perceptions regarding the management of aggressive behaviors in children and adolescents with ASD is defined as “consistent practices to smooth edges.”

“The aggressive behaviors displayed by primary school students with ASD are not serious or destructive; nonetheless their aggression needs to be addressed and controlled to prevent its progressive increase over time.”

(Focus Group 1; Primary school group)

“When I forced them to do something in terms of adjusting their behaviors, their explosive behavior remained unchanged.”

“Understanding and helping them to build trust with me seems to be rather more important, along with a consistent approach.”

(Focus Group 2; Middle school group)

### 3.2. Categories: Educational Responses to Individual Behaviors

#### 3.2.1. Identification of Aggressive Behavior Patterns

The management of aggressive behaviors in children and adolescents with ASD should be individualized according to the aggressive behaviors identified. Specifically, given that aggression in children and adolescents with ASD can vary widely from person to person, identifying aggressive behavior patterns at the individual level is the first step in treatment. This theme is further categorized into (1) characteristics of aggressive behaviors in children and adolescents with ASD, and (2) identification of the causes of aggressive behaviors. Aggressive behaviors of children and adolescents with ASD are manifested in four forms: verbal aggression (including yelling), aggression towards others, aggression toward self, and destruction of objects. These forms can appear individually or in combination, depending on the individual.

“There was a child who kept picking off a scab from a neck wound, delaying healing, and later tried to find a scab on someone else’s body to pick it off. This kind of behavior can take place without any malice.”

(Focus Group 1; Elementary School Group)

“Scratching, pinching, screaming, and running out; it’s really diverse.”

(Focus Group 1; Primary school group)

The participants indicated that the aggressive behaviors of students with ASD are largely provoked by: (1) desire to quickly obtain what they want, (2) desire to attract attention, (3) impulse to imitate or magnify what they see in their own homes, and (4) an unconscious desire to reduce anxiety and stress. Hence, it is agreed that aggressive behaviors are driven by the rebellious desire to get out of situations they do not want to be in, and can be interpreted as part of the expression needed to get what they want. It is also found that the presence of communication difficulties can increase the frequency of aggressive outbursts or tantrums. In particular, it is said that the intensity of aggressive behavior can rapidly increase when people respond to aggressive behaviors more quickly and intensively than to non-aggressive behaviors.

“Some children exhibit their aggression even when there is something they want to eat more of, or when they do not want to eat something their teacher recommends.”

(Focus Group 1; Primary school group)

“In general, children with low levels of verbal communication skills tend to be more aggressive. Their inability to express themselves clearly tends to trigger aggressive behavior.”

(Focus Group 3; High school group)

“One student who has some problems with speaking reacts sensitively to external stimuli (e.g., accidental touch) by yelling and crying, and their aggression gets worse. The student knows that such a reaction is more effective in drawing the teachers’ attention.”

(Focus Group 1; Primary school group)

#### 3.2.2. Strategic Responses to Aggressive Behaviors Observed

Strategic responses to the aggressive behaviors that appear in children and adolescents with ASD are divided into the following three types. 

The first response involves using physical seclusion immediately for the student displaying aggressive behaviors to help them calm down, and teachers can impose physical restrictions, if necessary, during this process. When there is a safety risk due to aggressive behavior, the participants’ responses were spontaneous and contained physical measures, and their responses included: (1) instructing the student concerned to stay apart from the group to help regain composure, (2) let other students leave the classroom to allow the student to stay alone, or (3) decide that all students can return to their home early. Special education schools have a dedicated comfort room used in these situations, while most general education schools do not have a similar space; thus, teachers physically seclude the student displaying aggressive behavior immediately, and help them regulate their behavior on site. In addition, once the student regains their composure after aggressive behavior, teachers guide the student to understand what is wrong with their behavior, and to take responsibility for the consequences (e.g., cleaning up the damaged area). Thus, spontaneous disciplinary measures are sought in the event of aggressive behaviors in schools.

“I let the student concerned take a break for 10 to 15 min in the comfort room or an empty classroom nearby without using physical restraint. I try to help the student calm down by turning off the light, playing music, or putting cushions, which are available in the comfort room. If secluding the student in a separate space is not possible, I let all the other students leave the classroom to enable the student to stay alone.”

(Focus Group 3; High school group)

The second strategy that teachers use in the event of aggressive behaviors is a gradual process in which the student concerned is continuously and repeatedly reminded that physical aggression should not be used as a means to reach a goal, and the teachers seek to improve psychiatric stabilization through continuous interaction with the student. In this regard, trust formation in the teacher–student relationship is essential, and teachers’ consistent attention and feedback is considered vital to successful outcomes. This gradual process may be considered time consuming and uncertain with respect to effectiveness. However, it is effective in reducing progressive aggression, and the participants considered this to be successful for behavioral adjustment if implemented consistently. Nonetheless, the need for a high degree of patience and teachers’ difficulties in slating disproportionately large amounts of time for one student pose limitations.

“I keep talking with the student to help them understand what’s happening around them and eventually to prevent aggression. I am repeatedly talking to nonverbal children.”

(Focus Group 1; Primary school group)

“Rapport building with teachers is important for them. I try to find the best way to effectively help children become stable is through continuous interactions. We sometimes count while breathing at the same pace, and I arrange activities that the student likes for specific days on which their aggression is predicted based on their behavioral history.

(Focus Group 2; Middle school group)

The third strategy is a preventive measure aimed at replacing or removing all environmental factors that could potentially trigger aggressive outbursts when the specific student is present. Many children are sensitive to light and sound and are prone to aggression as a result of changes in these two factors. Therefore, objects that may be obstacles to the preferred environment are removed as much as possible. Some of the participants take training programs or learn martial arts in order to safely engage with and restrain students who are physically aggressive. At the same time, other students in the same class are instructed to be aware of the possibility of aggressive behavior and to follow the teacher’s instructions if it actually occurs. The student concerned is also encouraged to take certain actions (e.g., pounding the table or clapping their hands) instead of taking aggressive actions such as hitting others with their hands.

“As a teacher at a special education school, I have received training and learned martial arts; I am a belt holder.”

(Focus Group 3; High school group)

“The student concerned is instructed to practice certain activities as an alternative to aggressive behaviors from the beginning of the semester, and tell them if you get an urge to take an action, clap hard and show it to me; this is my way of encouraging alternative behaviors.”

(Focus Group 2; Middle school group)

### 3.3. Categories: Experience in Interventions for Aggressive Behaviors

#### 3.3.1. Individual Intervention Practices

The participants were found to have experience in interventions for aggressive behaviors in children and adolescents with ASD, and considered a combination of drug and non-drug treatment as a necessary option. The participants themselves directly conduct interventions or sometimes collaborate with external parties when training programs or therapeutic interventions for students with ASD are outsourced. Given that drug treatment is not preferred by parents of the students concerned, due to possible adverse effects, the participants found it hard to convey their opinions on drug treatment when consulting with parents.

On the other hand, special education schools are equipped with physical restraints that can be used in the event of aggressive behaviors, although their use is minimized due to concerns over human rights infringement, while most general education schools do not have physical restraints. Therefore, most schools are more inclined to prevent aggressive behaviors by encouraging the students concerned to consume their energy through physical activities and exercises such as hiking.

“I encourage the student concerned to move a lot. Active activities such as walking around the school will make them feel better and help them stay out of trouble.”

(Focus Group 1; Primary school group)

“I sometimes encourage the student concerned to bring a sandbag to the school upon consent from their parents and we go hiking together to the mountain behind the school.”

(Focus Group 2; Middle school group)

“We highly recommend drug treatment. Parents want to see that their children do not need to take medicine. Indeed, there are side effects such as drowsiness and lethargy when drug treatment is used. I hope that specialized teachers can take part in the decision-making process for drug treatment.”

(Focus Group 3; High school group)

#### 3.3.2. School-Led Therapeutic Support

Currently, it is very rare for schools to prepare an aggression management manual for children and adolescents with ASD. In most schools, specialized teachers deal with the aggressive behaviors of students on their own and receive almost no support from their school even when they face problems during the course of their education. In general education schools, there are cases in which specialized teachers are called upon when a student with ASD causes trouble in an inclusive classroom, meaning that general educators do not share responsibility for students with ASD within a school. When the aggressive behaviors of students with ASD become an issue, and draw attention from the state educational administration office, remedial actions, such as outsourced consulting, are taken. However, no substantial level of effectiveness was observed in each case. The participants indicated that the most imperative need is an increase in teaching assistants. If each of them takes care of one student with autism, the overall management of the students with ASD would become much easier for specialized teachers, and it would eventually lower the prevalence of aggressive behaviors.

“There is no aggression management manual/guidelines prepared at school level. If there is any problem caused by a student with ASD in an inclusive classroom, the respective teacher calls me to pick up the student without any attempt to settle down the situation; they have no interest in the rationale for inclusive classroom.”

(Focus Group 2; Middle school group)

“There was a child who kept hitting their head on the bus, and a teaching assistant was assigned to the student to assist with their arrival at and departure from the school after the meeting with school representatives, parents, and other related personnel.”

(Focus Group 1; Primary school group)

#### 3.3.3. Factors Preventing Mitigation of Aggression

The participants underscored the importance of mitigating aggression in children and adolescents with ASD and claimed that efforts to mitigate aggression are lacking at home and school, which poses a barrier to the mitigation of aggression. Therefore, the participants stressed that solely specialized teachers and their efforts did not suffice to manage aggression in these students, and thereby called for the co-operation from all parties involved.

“Mitigation strategies would become less effective if family members and schools fail to demonstrate consistent educational approaches.”

(Focus Group 1; Primary school group)

“Selfish parenting style has a negative impact on aggression mitigation.”

(Focus Group 2; Middle school group)

### 3.4. Broader Theme: Acceptance to VR-Based Intervention Model for Aggression 

#### 3.4.1. Acceptance of VR-Based Program Application

The participants had a positive view of the application of VR-based intervention programs for aggression in children and adolescents with ASD based on the reason that interesting visual experiences and game-like features appeal to students. However, they cited students’ reluctance to wear a device when watching VR programs and teachers’ difficulty in maintaining the devices as barriers to using VR-based interventions. They did not rule out the possibility that students could become obsessed with VR programs due to the interesting content, and that such obsession could result in increased aggression.

“I think VR-based interventions would be a solution to overcome physical constraints felt in a school, and it would also help the students who keep breaking things because they will be satisfied by replicating such actions in a virtual space.”

(Focus Group 2; Middle school group)

“To watch VR-based programs, a device is required; nonetheless, it can be denied by some students.”

(Focus Group 1; Primary school group)

#### 3.4.2. Proposal for VR-Based Program Contents

We asked the interviewees, “Is there anything you would like to see included in a VR-based aggression reduction program?” This question was intended to provide useful information for content development for VR-based interventions designed to mitigate aggression in children and adolescents with ASD. The VR content that the participants thought was necessary is (1) environment-related content, (2) content offering a variety of sensory experiences, (3) content of social interaction development, and (4) others. 

First, environment-related VR content is meant to offer VR environmental experiences, especially those capable of promoting psychological stability. For example, the comfort room in a school or other familiar or stable environmental components can be employed as calming features. Such contents were expected to be particularly effective in inducing psychological comfort. Second, VR content offering multi-sensory experiences were thought to stimulate students’ interest. The participants further explained that being engaged in VR-based activities that students cannot experience in school (e.g., gardening and rock climbing) will help reduce aggressive behavior. Third, contents for the development of social interactions were suggested to be effective in teaching the basic knowledge required to fit society’s expectations through everyday life experiences, and to improve social skills. The fourth VR content suggested as “others” by the participants included training to improve the ability of understanding and interpreting others emotions, as this is generally lacking in students with ASD. Facial expression recognition training was also considered helpful in reducing aggression.

“They need something that enables them to express anger and relieve stress. Something like a heavy sandbag hung vertically so that they can hit it when they feel angry.”

(Focus Group 3; High school group)

“VR experiences in a variety of episodes involving aggressive behaviors can be a good practice; it is like a rehearsal for real-life situations involving the students themselves, and such situations can be reduced or prevented following VR experiences.”

(Focus Group 2; Middle school group)

## 4. Discussion

In this study, we explored specialized teachers’ perceptions of the management of aggressive behaviors in children and adolescents with ASD. The results revealed the core theme of “consistent practices to smooth edges” and three categories. The first category, “educational responses to individual behaviors” had the sub-themes of “identification of aggressive behavior patterns” and “strategic responses to aggressive behaviors.” The second category, “experience in interventions for aggressive behaviors” had the sub-themes of “individual intervention practices”, “school-led therapeutic support”, and “factors preventing mitigation of aggression.” The third category, “acceptance of VR-based intervention model for aggression in children and adolescents with ASD” had the sub-themes of “acceptance of VR-based program applications” and “proposal for VR-based program content.”

With regard to the core theme, the participants stressed the need for a consistent and constant treatment approach, to mitigate and eliminate aggressive behaviors in children and adolescents with ASD; while physical aggression is not ubiquitous in these students, it tends to worsen when it is present. They highlighted the importance of consistent interventions based on trust in the teacher–student relationship, which supports the findings of previous studies that report the important impact of specialized teachers’ individualized and consistent educational approaches [11,12,13,14]. 

Regarding the first category, the participants perceived that educational responses to aggressive behaviors need to be individualized according to the behavioral patterns identified, and based on the characteristics and causes of aggression. This result is consistent with a previous study in which the behavioral patterns of children and adolescents with ASD were identified and used as the basis for individualized intervention applications [10]. Strategic responses to aggressive behavior situations identified were perceived as: (1) spontaneous with the aim of avoiding safety accidents; (2) consistent, repetitive, and gradual; and (3) preventive, through the removal of possible stimulators. These results are in agreement with the existing literature in which environmental components have been reported as important, in addition to teachers’ education [11,12,23]. However, ASD shows functional differences according to the severity level, and the level of support also varies from level 1 (“support”) to level 3 (“very substantial support”) dependingly. In addition, it may affect the pattern and degree of aggressive behaviors in ASD [1,24]. Therefore, considering the severity level of children and adolescents with autism spectrum disorder, it is necessary to further subdivide and apply the response methods classified into the three categories. In particular, considering that special schools and special classes in general schools (both mainstream and special education) differ greatly in terms of educational management, and the severity level of students who attend special schools is higher, the educational response policy, according to the type of school, must be established and applied differently.

In discussion of the second category, the participants talked about their own experiences in interventions for aggressive behavior and considered a combination of drug and non-drug treatment approaches necessary. They also used physical activities as the most common non-drug intervention, which coincides with the findings of previous studies reporting the beneficial effect of physical activities on aggressive behavior in children and adolescents with autism [25]. Additionally, the participants described a lack of school-led guidelines, absence of joint responsibility, and the insufficient number of teaching assistants as problems at the school level. They concluded that the lack of perception of the need for mitigation of aggression, and associated efforts, acts as an obstacle. This result is consistent with previous studies that emphasized the application of intervention programs that involved family members, friends, and other people who closely interact with individuals with autism [23,26]. 

In the third category, the participants considered the use of VR-based interventions for aggressive behaviors to stimulate their students’ interest. Earlier research also showed that VR experiences would be more interesting than real-life situations [17,18]. However, they were concerned about the maintenance of VR devices and the possibility of addiction. As for the content of VR-based interventions for aggressive behaviors in students with ASD, the participants focused on environment, sensory experiences, and social interaction development. This finding agrees with earlier research reporting that VR-based training of children and adolescents with ASD is effective in improving their social skills, such as empathy and gaze direction [17,19], and that augmented reality-based interventions are successful [27,28]. Telerehabilitation intervention reduces problem behaviors and improves the adaptation ability of children and adolescents with multiple disabilities [29]. Thus, the importance of developing active VR contents or telerehabilitation (TR) programs with new technologies is suggested.

This study has several limitations. First, it explored specialized teachers’ perceptions of the management of aggressive behaviors in children and adolescents with ASD, and their understanding and viewpoints on aggressive behavior may differ from those of the children and adolescents with autism and their parents. Accordingly, further qualitative studies using in-depth interviews are necessary to gain insights into these families regarding the management of aggressive behaviors. Second, specialized teachers’ experiences may be affected by multiple severity levels in children and adolescents with ASD. Based on the findings of focus group interviews, a quantitative study needs to be conducted to identify teachers’ different perception levels regarding the management of aggressive behaviors in children and adolescents with autism. In this study, all the interviews were conducted in Korean, and interview data were translated into English. Therefore, the possibility of mistranslation cannot be ruled out. The translated version was proofread and edited to achieve maximum translation accuracy. 

## 5. Conclusions

As key elements in their perceptions regarding aggression management, specialized teachers suggested that the aggressive behaviors of children and adolescents with ASD should be mitigated or eliminated under a consistent and long-term approach. It was also found that the management of physical aggression should start with the identification of behavioral patterns and that educational responses should be adapted to the individual characteristics identified. With regard to experiences in interventions for aggressive behaviors, school support is imperative for teachers and their intervention practices. More importantly, the combined efforts of all parties involved are crucial to improve aggression mitigation. The use of VR-based interventions was perceived positively, and contents on environment, social interaction development, and other areas were recommended. Considering these findings, the following suggestions are made. First, further in-depth studies are needed to explore the perceptions of children and adolescents with autism and their parents, and the programs they prefer, with respect to the management of their aggressive behaviors. Second, the insights into special education required in Korea and the development of VR-based intervention content should be further investigated. Finally, further studies are also necessary to track the development of VR-based education and training programs and subsequent applications by specialized teachers as intervention strategies for aggression in students with ASD.

## Figures and Tables

**Table 1:** Participants’ characteristics.

Group	ID (N = 13)	Teaching Experience		Gender	Affiliation Address		General or Special Education School	
		yr	Mean ± SD		Name of City (Province)	N (%)	Type	N (%)
Group 1 (Primary school teacher)	Participant A	17	9.27 ± 4.94	F	Seoul	Gyeonggi province 6 (46.15%) Incheon 1 (7.70%) Seoul 6 (46.15%)	General	General 10 (76.92%) Special 3 (23.08%)
	Participant B	4		F	Seoul		Special	
	Participant C	7		F	Gyeonggi province		Special	
	Participant D	12		F	Gyeonggi province		General	
Group 2 (Middle school teacher)	Participant E	1		F	Gyeonggi province		General	
	Participant F	10		F	Seoul		General	
	Participant G	11		F	Gyeonggi province		General	
	Participant H	13		F	Gyeonggi province		General	
Group 3 (High school teacher)	Participant I	5		F	Incheon		General	
	Participant J	2.5		F	Gyeonggi province		General	
	Participant K	15		F	Seoul		General	
	Participant L	11		F	Seoul		General	
	Participant M	12		F	Seoul		Special	
